# Harnessing Gene Conversion in Chicken B Cells to Create a Human Antibody Sequence Repertoire

**DOI:** 10.1371/journal.pone.0080108

**Published:** 2013-11-21

**Authors:** Benjamin Schusser, Henry Yi, Ellen J. Collarini, Shelley Mettler Izquierdo, William D. Harriman, Robert J. Etches, Philip A. Leighton

**Affiliations:** 1 Department of Animal Science, University of California Davis, Davis, California, United States of America; 2 Crystal Bioscience Inc., Emeryville, California, United States of America; National Cancer Institute, NIH, United States of America

## Abstract

Transgenic chickens expressing human sequence antibodies would be a powerful tool to access human targets and epitopes that have been intractable in mammalian hosts because of tolerance to conserved proteins. To foster the development of the chicken platform, it is beneficial to validate transgene constructs using a rapid, cell culture-based method prior to generating fully transgenic birds. We describe a method for the expression of human immunoglobulin variable regions in the chicken DT40 B cell line and the further diversification of these genes by gene conversion. Chicken V_L_ and V_H_ loci were knocked out in DT40 cells and replaced with human V_K_ and V_H_ genes. To achieve gene conversion of human genes in chicken B cells, synthetic human pseudogene arrays were inserted upstream of the functional human V_K_ and V_H_ regions. Proper expression of chimeric IgM comprised of human variable regions and chicken constant regions is shown. Most importantly, sequencing of DT40 genetic variants confirmed that the human pseudogene arrays contributed to the generation of diversity through gene conversion at both the *Igl* and *Igh* loci. These data show that engineered pseudogene arrays produce a diverse pool of human antibody sequences in chicken B cells, and suggest that these constructs will express a functional repertoire of chimeric antibodies in transgenic chickens.

## Introduction

Monoclonal antibodies (mAB) are an important pillar in the treatment of multiple disorders such as cancer, inflammatory diseases, and orphan diseases [1]–[3]. With the development of hybridoma technology, it became possible to produce mAB in mice [4]. Because of their murine origin, however, these antibodies are immunogenic in humans [5], [6]. To reduce immunogenicity, chimeric antibodies, humanized antibodies and fully human antibodies from phage display libraries were created using recombinant DNA techniques [7]–[10]. Another attempt to solve this problem was to create transgenic animals carrying human immunoglobulin loci in order to produce human sequence antibodies directly without further manipulation [11]–[15]. The animal-based approaches are all limited by the fact that some antigens, especially human tumor antigens, are not well recognized in mammals because of the close evolutionary relationship to humans. To date, all of the transgenic animals producing human antibodies have been mammalian species, but a non-mammalian host such as chicken would access a much wider set of epitopes, since chickens have not shared a common ancestor with humans in at least 300 million years. The complex genetic modifications necessary to produce human antibodies in chickens (knockout of endogenous immunoglobulins and insertion of human transgenes) can be accomplished in cultured primordial germ cells, leading to the creation of fully transgenic birds. [16], [17].

The chicken B cell line DT40 expresses a normal surface IgM receptor and continues to diversify its immunoglobulin loci by the process of gene conversion, a type of homologous recombination [18]. Gene conversion generates sequence diversity in the functional light and heavy chain variable regions by using upstream pseudogenes as the sequence donors in a template-driven, unidirectional process to mutate the single rearranged V region in each locus [19]. Wild type DT40 cells have been used to generate antigen-specific antibodies from the endogenous immunoglobulin loci in vitro but the variable regions remained chicken sequence [20]–[22]. The ability of DT40 cells to promote gene conversion has been applied to exogenous genes such as GFP, which was inserted into the immunoglobulin light chain locus [23], [24]. The application of gene conversion to exogenous genes requires that the gene of interest be inserted in an immunoglobulin locus, as the gene conversion machinery preferentially acts at these loci over other loci [25]–[27], and it requires that pseudogenes be present to serve as sequence donors. Although the DT40 gene conversion machinery could be used directly for the diversification of human immunoglobulin variable regions that could be used in antibody discovery programs, we believe an *in vivo* immune system with affinity maturation will generate higher affinity antibodies with higher efficiency [28]. However, DT40 cells can still serve an important role in validating transgene constructs prior to insertion into transgenic chickens.

Here, we demonstrate production of a repertoire of human V region sequences by gene conversion, using a DT40 cell line with a double knock out of the chicken immunoglobulin light (*Igl*) and heavy (*Igh*) chain loci. The double knock-out cell line was used for a targeted, site-specific integration of functional human V_K_ and V_H_ genes, thereby replacing the chicken functional V_L_ and V_H_ genes (*Igl^huVK^, Igh^huVH^*). In order to diversify the inserted huV_K_ and huV_H_ by gene conversion, we included synthetic human sequence pseudogene arrays upstream of the functional huV_K_ and huV_H_. By using a stop-codon reversion assay we show that *Igl^huVK^, Igh^huVH^* DT40 cells diversified the functional human heavy and light chain genes by gene conversion, suggesting that these transgenes, when inserted into fully transgenic chickens, will create a diverse repertoire of human antibodies in B cells *in vivo*. This demonstration of simultaneous *in vitro* molecular evolution of two genes in the same cell line can be generalized to provide a method for creating libraries of proteins whose sequence are defined by the pseudogene arrays.

## Materials and Methods

### Cell Culture

DT40 cells were a generous gift from Sherie L. Morrison (Department of Microbiology, Immunology & Molecular Genetics, University of California, Los Angeles, USA) [29]. DT40 cells were cultured at 37°C in IMDM (Life Technologies, Carlsbad, USA) supplemented with 10% fetal bovine serum, 1% chicken serum, 100 µM 2-mercaptoethanol and 0.5% Penicillin/Streptomycin.

### Transfection of DT40 Cells

For each transfection, 5×10^6^ cells were collected, pelleted and resuspended in V-buffer (Lonza Walkersville Inc., Walkersville, USA) with 10 µg DNA of each construct for a total volume of 100 µl. The cell-DNA suspension was transferred to a 2 mm cuvette and subjected to 8 square wave pulses of 350 V/125 µsec (BTX 830 electroporator). The transfected cells were resuspended in medium and plated into a 96 well plate. Selection with either 0.5 µg/ml puromycin, 5 mg/ml neomycin, 100 µg/ml blasticidin, or 2 mg/ml hygromycin was started 24 hours following transfection. As soon as single colonies were identified they were transferred to new wells and expanded for further analysis.

### Construction of the Puromycin/Neomycin/eGFP Selectable Marker Cassette

The enhanced green fluorescent protein (eGFP) driven by the chicken β-actin promoter was cloned with the puromycin-resistance gene driven by the CAG promoter. The eGFP-puro cassette was flanked by two sets of duplicated HS4 insulators. A 46 bp attP site (GTGCCCCAACTGGGGTAACCTTTGAGTTCTCTCAGTTGGGGGCGTA) was cloned upstream of a promoterless neomycin resistance gene using annealed oligos, and a 34 bp loxP site (ATAACTTCGTATAGCATACATTATACGAAGTTAT) was inserted downstream of the neo gene using annealed oligos. The attP-neo-loxP was then inserted into the eGFP-puro cassette. A second loxP site was added at the 5′ end of the cassette by ligating annealed oligos to restriction enzyme-digested plasmid. A sequence from HA (AAGCGTAATCTGGAACATCGTATGTA) was also included as a primer binding site for the PCR genotyping assay.

### Construction of the Hygromycin/Blasticidin Selectable Marker Cassette

A hygromycin resistance gene driven by the chicken β-actin promoter was obtained from H. Arakawa (plasmid 257) and cloned in between two sets of duplicated HS4 insulators from the chicken β-globin locus. The promoterless blasticidin resistance gene (Bsr) was amplified from a Bsr-containing plasmid (597, a gift from H. Arakawa) using primers that included a 51 bp attP site 5′ of the Bsr gene, and a 34 bp loxP site at the 3′ end. (Primers: 5′-TTACGTAGTGCCCCAACTGGGGTAACCTTTGAGTTCTCTCAGTTGGGGGCGTAGGTCATTTTTGCAGAAATCGGAGGAAG-3′ and 5′-CCATGCATATAACTTCGTATAGCATACATTATACGAAGTTATGGATCCAGACATGATAAGATACA-3′).

A second loxP site was added at the 5′ end of the cassette by ligating annealed oligos to restriction enzyme-digested plasmid. The HA sequence from above was also included as a primer-binding site for the PCR genotyping assay in transfected cells.

### Construction of the Chicken V_L_ Targeting Constructs

The 5′ homology region (1 kb) was amplified from chicken genomic DNA using primers:

5′-GCCCATCACTCAGGGAGGAGA-3′

5′-TAGCTCTGAAGTCTCCATCCT-3′

The unique restriction sites AscI and NheI were used to clone the 5′ homology region upstream of the selectable marker cassette

The 3′ homology region (7.2 kb) was amplified from chicken genomic DNA in two steps, and the products were combined by ligation via a naturally occurring BssSI restriction site. The 5′ and 3′-most primers delineating the 3′ homology region are:

5′-CTGAACTAGTGCTGACTCTGCA-3′

5′-GCCGCACGTTGCACAGCTGT-3′

The unique restriction sites SpeI and NotI were used to clone the 3′ homology region downstream of the selectable marker cassette.

### Construction of the Chicken V_H_ Targeting Construct

The 5′ homology region (1.1 kb) was amplified from DT40 genomic DNA using primers:

5′-CTCAGAGCCCCTAATAAGTG-3′

5′-TCTGCGCTGAGTTCTTTGA-3′

The unique restriction sites AscI and NheI were used to clone the 5′ homology region upstream of the selectable marker cassette.

The 3′ homology region (4.3 kb) was amplified from DT40 genomic DNA using primers:

5′-TGGCGGTGTAGGGGAAAATGTC-3′

5′-AGCCCCTAATAACCGTAAT -3′

The unique restriction sites SpeI and NotI were used to clone the 3′ homology region downstream of the selectable marker cassette.

### Knock Out of Chicken V_L_ and V_H_

**Figure 1:**
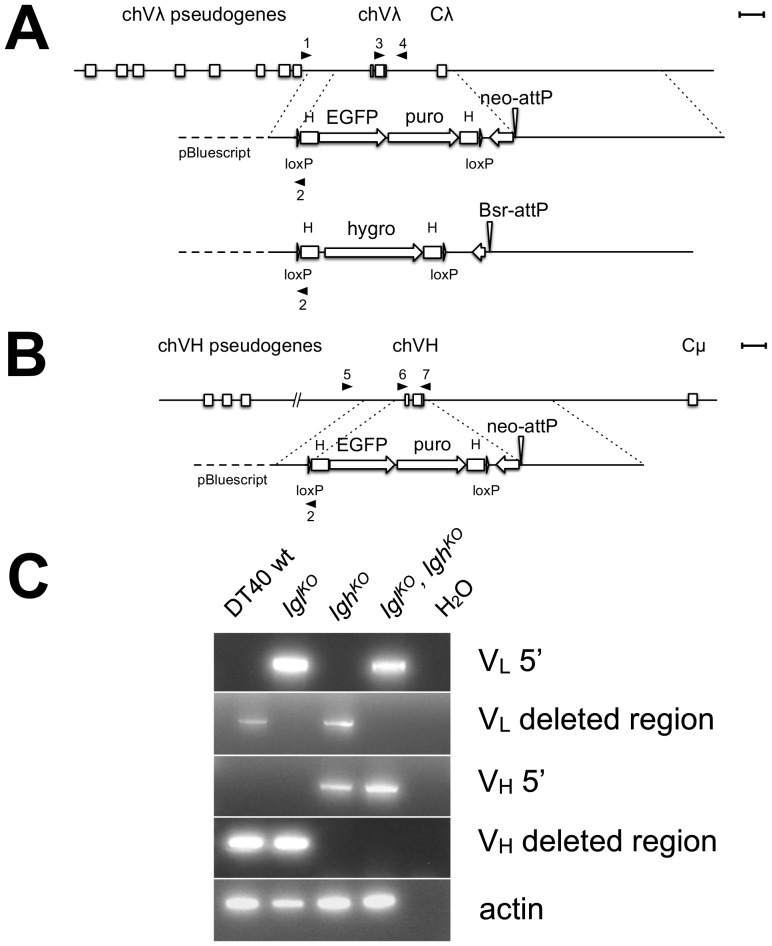
Knockout of the chicken V_L_ and V_H_ genes. Chicken V_L_ and V_H_ loci are shown and the homology regions of the knockout constructs flanking the deleted region are marked by dotted lines. Targeting constructs for the V_L_ and V_H_ in chicken DT40 cells are shown. a) For the V_L_ single knockout, the knockout construct consists of a β-actin-eGFP (eGFP), a CAG-puromycin (puro) selectable marker cassette and the 5′ and 3′ homology regions. To create the double knockout of V_L_ and V_H_, an alternative targeting construct for V_L_ has a β-actin-hygromycin (hygro) selectable marker cassette. The homology regions are the same for both V_L_ knockout constructs. b) For the V_H_ knockout, the targeting construct consists of a 5′ and 3′ homology region, a β-actin-eGFP and a CAG-puromycin selectable marker cassette. In all constructs, the selectable markers are flanked by the HS4 insulator from chicken beta-globin (H), and loxP sites [39]. Downstream of the 3′ loxP site a promoterless neomycin (neo) or blasticidin (Bsr) gene in opposite orientation together with an attP site for targeted insertion is included. c) Knockout of the V_L_ and V_H_ was detected with gene specific primers on the 5′ side as well as with primers in the deleted region (black arrow heads: VL 5′, primers 1 and 2; VL deletion region, primers 3 and 4; VH 5′, primers 5 and 2; VH deleted region, primers 6 and 7). β-actin served as a quality control for the genomic DNA. Scale bar equals 1 kb.

To create the *Igl^KO^* cell line with a knockout of the chicken V_L_, DT40 cells (see above) were transfected with the EGFP-puro containing construct shown in Fig. 1a and single colonies were selected for puromycin resistance and eGFP expression. To create the *Igh^KO^* cell line with a knockout of the chicken V_H_, cells were transfected with the targeting construct shown in Fig. 1b and single colonies were selected for puromycin resistance and eGFP expression. To create the *Igl^KO^*, *Igh^KO^* cell line with a double knockout of V_L_ and V_H_, a stable pool of DT40 *Igh^KO^* cells was transfected with the alternative V_L_ targeting construct containing the hygro gene shown in Fig. 1a and single colonies were selected for hygromycin/puromycin resistance and eGFP expression. Clonal populations were expanded after every selection and genomic DNA was isolated using DNeasy Blood&Tissue Kit (Qiagen, Valencia, USA). FIREPol PCR Mix 7.5 (Solis BioDyne, Tartu, Estonia) was used according to the manufacturer’s protocol with the following primers to verify the correct targeting and deletion of the V_L_ and V_H_ regions.

V_L_ knockout 5′ targeting forward primer: 5′-ACTGTGCTGCAGGTGGCTATG-3′ (primer #1), V_H_ knockout 5′ targeting forward primer: 5′-TGGTTTGGTTGATGGAAGAATGTA-3′ (primer #5), V_L_ and V_H_ knockout 5′targeting reverse primer in HA: 5′-ATACGATGTTCCAGATTACGCTT-3′ (primer #2), V_L_ deleted region: 5′-GAGACGAGGTCAGCGACTCAC-3′ (primer #3), 5′-GGCTGCGATCGCCGCGCTGACTCAGCCGTCCTC-3′ (primer #4), V_H_ deleted region: 5′-ATGGCGGCCGTGACGTTGGA-3′ (primer #6), 5′-CGGAGGAGACGATGACTTCGG-3′ (primer #7).

### Selection of Functional huV_K_ and huV_H_

The functional human V_K_ and V_H_ genes were selected after screening a small library of genes from the V_K_3 and V_H_3 gene families cloned from human B cell DNA for a V_K_/V_H_ pair that would express at high levels in HEK293 cells. A V_K_3–15 gene and a V_H_3–23 gene were chosen as the functional variable regions for expression in DT40. The sequences of the V_K_ and V_H_ functional genes served as the starting template for the design of the human pseudogenes.

### Construction of the SynVK Insertion Constructs

For the SynVK-12 pseudogene array, huV_K_ pseudogenes were designed based on the DNA sequence encoding the mature huV_K_3–15 polypeptide from the signal peptide cleavage site to the CDR3 region without any JK sequence. Framework regions (FW) and CDRs were defined using the IMGT domain system [30]. Each position in CDR1, CDR2 and the first 6 positions of CDR3 were individually substituted with either Tyrosine (Y) or Tryptophan (W), and the FW are identical to the functional huV_K_ gene. Each pseudogene contains at most one amino acid change in each of its three CDRs.

For the SynVK-C array, the sequence of the functional huV_K_ gene was altered to incorporate AID hotspots throughout the V region (nucleotides WRC/GYW) without changing the amino acids encoded. The pseudogenes, where homologous to the functional huV_K_, contained the same changes to maintain the homology. For the pseudogene array, variable region sequences were obtained from the NCBI human EST database and a diverse set of CDR1, CDR2 and CDR3 sequences were selected. FW diversity was also included in some of the pseudogenes, while the remaining pseudogenes contained FW identical to the functional V_K_. FW and CDRs from different ESTs were assembled together into the pseudogenes, such that each pseudogene could be a combination of 3–6 EST sequences. Spacer sequences (100 bp between each pseudogene) were derived from the chicken ΨV_L_ pseudogene locus. The pseudogenes were synthesized (BioBasic, Markham, Ontario, Canada) as individual genes and ligated together sequentially into the array. The pseudogenes were all in a direct repeat orientation (except for the most distal pseudogene in SynVK-12), and in the opposite orientation relative to the functional huV_K_. Plasmids containing the huV_K_ pseudogene arrays were propagated in HB101 cells (MCLab, South San Francisco, USA) for stability.

The remainder of the light chain locus in the SynVK insertion vectors was derived from the chicken light chain by PCR. The chIgL promoter, J-C intron, and Cλ constant region were assembled with the rearranged human V_K_ gene and the human leader exon. The chicken leader intron was included in the gene synthesis of the human V_K_ gene. The human pseudogene array was cloned upstream of the 2.4 kb promoter fragment, putting the most proximal pseudogene in a position nearly identical to that in the endogenous light chain locus. For insertion into the attP site at the knockout locus, an attB site (either 45 or 70 bp) was included adjacent to the chicken β-actin promoter that will drive expression of the promoterless blasticidin or neomycin genes in the knockout. The HS4 insulator was included upstream of the β-actin promoter.

### Construction of the SynVH Insertion Constructs

The SynVH pseudogenes were designed based on the functional huV_H_ gene, and extend from the start of the mature protein to the CDR3 sequence, without any JH sequence. For the SynVH-B pseudogene array, all FW were identical to the functional huV_H_, and the CDRs contained individual substitutions of Y, W or Serine (S) residues. Each CDR in each pseudogene has a single amino acid substitution relative to the functional huV_H_. Several pseudogenes contained CDRs identical to the functional V_H_ because of naturally occurring Y, W or S residues in those positions. Spacer sequences between pseudogenes (100 bp) were obtained from the chicken ΨV_H_ pseudogene locus. The pseudogenes were synthesized and cloned into an array by sequential rounds of ligation.

The non-coding sequences (2.1 kb chicken V_H_ promoter, and 275 bp of the chicken J-C intron) were amplified from chicken genomic DNA and assembled with the rearranged human V_H_ region and human leader exon. The chicken V_H_ leader intron was included in the gene synthesis of the human functional V_H_. The SynVH-B array was then cloned upstream of the chV_H_ promoter, with the pseudogenes all in opposite orientation to the functional huV_H_. The β-actin-attB cassette for selection of insertions was the same as in the SynVK insertion vectors.

For the SynVH-A7 array, the pseudogenes have the same basic structure as those in the SynVH-B array, with sequences extending from the start of the mature protein coding sequence to the end of CDR3, and similar spacer sequences between the pseudogenes. Every CDR is composed entirely of varying combinations of Y, W and S residues. Whereas the CDRs in the SynVH-B array had single substitutions in each CDR, the CDRs in the SynVH-A7 array had all positions substituted. The FW are identical to the functional huV_H_, and the remainder of the construct is identical to the SynVH-B insertion vector.

### Integrase-mediated Insertion of huV_K_ and huV_H_

For insertion of the huV_K_ and huV_H_ constructs, *Igl^KO^* and *Igh^KO^* single knockout cell lines (see above) were transfected as described above with 10 µg ΦC31 integrase and 10 µg of the huV_K_-SynVK-12, huV_K_-SynV_K_-C, huV_H_-SynV_H_-B or huV_H_-SynV_H_-A7 construct. 24 hours after transfection G418 selection was started. Integration of the huV_K_ and huV_H_ constructs was tested by PCR on genomic DNA samples from the transfected cells. To verify the integration on the 5′ side for all of the single insertion constructs, the primers 5′-CTCTGCTAACCATGTTCATGCCTTC-3′ (primer #9) and 5′-AGTGACAACGTCGAGCACAGCT-3′ (primer #12) were used. The integration on the 3′ side was confirmed for the huV_K_ using the primers 5′-CGCACACGTATAACATCCATGAA-3′ (primer #10) and 5′-GTGTGAGATGCAGACAGCACGC-3′ (primer #11) and for the huV_H_ 5′-TTTGACTACTGGGGCCTGG-3′ (primer #13) and 5′-GCCCAAAATGGCCCCAAAAC-3′ (primer #14). To generate a cell line expressing both huV_K_ and huV_H_, *Igl^KO^, IgH^KO^* double knockout cells (see above) were first transfected with 10 µg huV_H_-SynV_H_-B and 10 µg ΦC31 integrase. After selection for neomycin/puromycin/hygromycin resistance and eGFP expression, the integration of the huV_H_-SynV_H_-B construct was confirmed by the same PCR used for the single insertion of the huV_H_. These cells were then transfected with 10 µg ΦC31 integrase and 10 µg of the huV_K_-SynV_K_-12 or huV_K_-SynV_K_-C construct. 24 hours after transfection cells were selected for neomycin/puromycin/hygromycin/blasticidin resistance and eGFP expression. The resulting cell lines were tested for the integration of the huV_K_ construct and re-confirmed for the integration of the huV_H_-SynV_H_-B construct as above. Because the selectable marker cassette used for the huV_K_ construct differs from the one used for the single integration of the huV_K_ a different primer set was needed to verify the 5′ integration: 5′-AATTGCCGCTCCCACATGATG-3′ (primer #8) and 5′-CTCTGCTAACCATGTTCATGCCTTC-3′ (primer #9).

### Generating eGFP Negative Cell Lines

In order to create the eGFP-negative *Igl^KO^, Igh^KO^* and *Igl^huVK^,Igh^huVH^* cell lines, cells were transfected with 10 µg pCAG-Cre (gift from Connie Cepko, Harvard Medical School, Boston, USA) by electroporation. Cells were monitored for the loss of eGFP expression over several days following the transfection and as soon as non-green cells were observed, 50 cells were plated on a 96 well plate to obtain single cell clones. Non-green clones were identified after days and were expanded for further study.

### Western Blot

1×10^6^ DT40 cells were lysed in 50 µl 1x Lysing buffer (Cell Signaling, Danvers, USA) according to the manufacturer’s protocol. 10 µl of the cell lysate were mixed with 4x LDS sample buffer (Life Technologies, Carlsbad, USA) and reducing agent (Life Technologies, Carlsbad, USA) to a final concentration of 1x and heated for 5 min at 95°C. Samples were separated by SDS-PAGE. Cµ protein was detected by a polyclonal anti-chicken IgM antibody conjugated with alkaline phosphatase (Bethyl Laboratories Inc., Montgomery, USA). V_L_ was detected by a polyclonal rabbit anti-chicken-IgY antibody conjugated with alkaline phosphatase (Sigma Aldrich, St. Louis, USA). β-actin was detected by a monoclonal β-actin antibody (Novus Biologicals LLC, Littleton, USA) followed by a polyclonal anti-mouse-IgG antibody conjugated to alkaline phosphatase (Jackson Immunoresearch, West Grove, USA). Alkaline phosphatase substrate (Life Technologies, Carlsbad, USA) was used for visualization.

### Flow Cytometry Analysis

1×10^6^ cells were pelleted and washed with cold labeling buffer (PBS containing 0.5% bovine serum albumin). Cells were resuspended in 500 µl cold labeling buffer containing a monoclonal anti-chicken-IgM antibody or polyclonal anti human-kappa-RPE antibody (Southern Biotech, Birmingham, USA). Cells were incubated for 30 min in the dark at 4°C. After washing two times with cold labeling buffer the anti-chicken-IgM antibody was detected by a polyclonal anti-mouse-IgG antibody (Bethyl Laboratories Inc, Montgomery, USA) conjugated to Cy5. After incubation for another 30 mins, the cells were washed twice with cold labeling buffer and resuspended in labeling buffer. Fluorescence was measured using a Beckman-Coulter FC500.

### Ca^2+^ Signaling

1×10^6^ cells were washed with labeling buffer (calcium/magnesium free PBS containing 2% fetal bovine serum and 1 g/L glucose) at room temperature. Cells were then resuspended in labeling buffer and 0.5 µM FLUO-4 (Life Technologies, Carlsbad, USA) and incubated for 20 min in the dark at room temperature. Afterwards cells were washed three times with the labeling buffer and resuspended in 500 µl labeling buffer. Background fluorescence was measured for 20 sec on a Beckton Dickinson LSRII, Fortessa followed by cross-linking the immunoglobulin receptor with a polyclonal anti-chicken-IgM antibody at a final concentration of 10 mg/ml. Change in fluorescence was measured for additional 220 sec.

### Amplification and Cloning of Gene-converted huV_K_ and huV_H_ Regions

To select for gene-converted sequences by PCR, a reversion assay was designed. A HpaI restriction site, containing a stop codon, was included in CDR1 or CDR3 of the transcribed huV_K_ and huV_H_ transgenes. These constructs were otherwise identical to those described above. DT40 cells were tranfected with these constructs as described above, resistant clones were confirmed by PCR, and the huV_K_ and huV_H_ sequences were analyzed after growing these cells at least four weeks to allow accumulation of mutations. Genomic DNA was isolated using the DNeasy Blood&Tissue Kit (Qiagen, Valencia, USA) according to the manufacturer’s protocol. Genomic DNA was digested with HpaI over night. After digestion, the huV_K_ region was amplified using primers 5′-ACTGCACCGGAGAAATTGTCTTG-3′ and 5′-GAGACGAGGTCAGCGACTCAC-3′. The huV_H_ region was amplified using primers 5′-GACGTGCAGTTGGTGGAGTC-3′ and 5′-GTTGAAGACTCACCTGAGGAGACGG-3′. The PCR was performed using GoTaq (Promega, Madison, USA) according to the manufacturer’s protocol. The PCR product was again digested with HpaI and the remaining product was gel purified using the Ultra Sep Gel Extraction Kit (Omega Bio-tek, Norcross, USA) according to the manufacturer’s protocol. The purified product was cloned into pCR2.1 using the TOPO/TA cloning kit (Life Technologies, Carlsbad, USA). 96 w plasmid preparations were performed using the Wizard SV 96 Plasmid Purification System (Promega, Madison, USA). Isolated DNA was sent for sequencing (MCLab, South San Francisco, USA).

### Analyzing Gene Conversion Events

A reference database of all human CDRs and framework regions used in the pseudogenes in the different arrays was created. Sequences from the amplified huV regions were aligned using Lasergene (DNAStar Inc., Madison, USA) and exported to a FASTA database. These two databases were compared by using CD-HIT [31], [32] to identify full-length gene conversion events. All shorter events, point mutations and deletions were verified manually.

## Results

### Knock Out of Chicken V_L_ and V_H_ in the Chicken B Cell Line DT40 Results in Loss of Surface IgM Expression

To replace the rearranged chicken V_L_ and V_H_ in DT40 cells with functional huV_K_ and huV_H_, the chicken genes must first be knocked out. Targeting constructs for the V_L_ and the V_H_ were designed (Fig. 1a and b). The constructs consisted of a selectable marker cassette and 5′ and 3′ homology regions. The homology regions for the V_L_ targeting constructs were 1.1 kb on the 5′ side and 7.2 kb on the 3′ side (Fig. 1a). For the V_H_ targeting construct, the homology regions were 1.1 kb on the 5′ side and 4.3 kb on the 3′ side (Fig. 1b). To generate cell lines with a single knockout of either *Igl* or *Igh*, the selectable markers consisted of a puromycin resistance gene and an eGFP gene. For double knock-out *Igl^KO^, Igh^KO^* cell lines, a hygromycin resistance cassette was used in the V_L_ targeting construct. No eGFP was included since the V_H_ targeting construct already expresses eGFP (Fig. 1b). The selectable marker cassettes also included an attP site for later site-specific integration using ΦC31 integrase of an insertion construct carrying an attB site. Linked to the attP site was a promoterless drug-resistance gene, either neomycin or blasticidin, in opposite orientation. By inserting a promoter into the attP site in front of this drug-resistance gene, clones with the correct insertion can be selected with the appropriate drug selective media. All cassettes were flanked by loxP sites to loop out the selectable markers by treatment with Cre recombinase. After transfection with the single targeting constructs, clonal populations were selected and analyzed for the correct targeting by PCR (Fig. 1c). Primers binding in the deleted region can also bind on the unrearranged allele but since the PCR extension time was kept short no PCR product was observed. The targeting efficiency for both the chicken V_L_ and V_H_ was ∼15%. The chV_H_ knock-out cell line (*Igh^KO^*) was transfected with the alternative V_L_ targeting construct to obtain a double knock out of V_L_ and V_H_ (*Igl^KO^, Igh^KO^*) in the same cell line. After selecting these cells with puromycin/hygromycin, the double knock out was confirmed by PCR (Fig. 1c). The targeting efficiency here was ∼10%.

**Figure 2:**
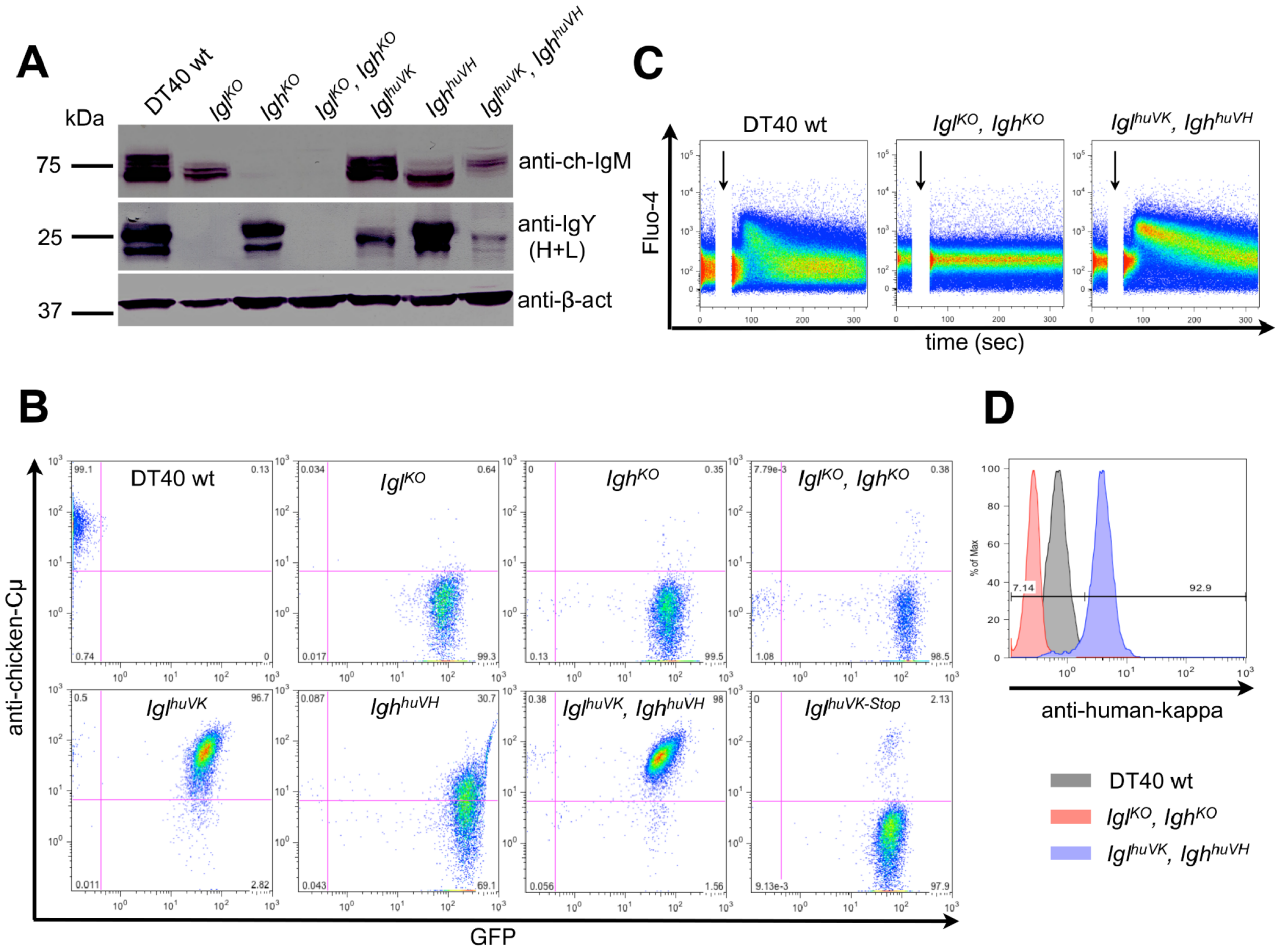
Expression and signaling in DT40 cells with immunoglobulin knockouts and chimeric immunoglobulin insertions. Wild-type cells (DT40 wt), V_L_ knockout (*Igl^KO^*), V_H_ knockout (*Igh^KO^*) and V_L_-V_H_ double knockout cells (*Igl^KO^, Igh^KO^*) as well as huV_K_ insertion (*Igl^huVK^*), huV_H_ insertion (*Igh^huVH^*) and huV_K_-huV_H_ (*Igl^huVK^, Igh^huVH^*) double insertion cell lines were analyzed for expression and signaling of immunoglobulin receptors. a) 1×10^6^ cells were lysed and immunoglobulin heavy chain expression determined by Western blotting using goat-anti-chicken-IgM-AP and immunoglobulin light chain expression by rabbit-anti-chicken-IgY-AP. Mouse-anti-β-actin followed by goat-anti-mouse-AP was used to detect β-actin. b) The cell lines from above and *Igl^huVK^* cells with a stop codon (*Igl^huVK-Stop^*) in CDR1 cultured for four weeks were stained with mouse-anti-chicken-IgM followed by goat-anti-mouse-Ig-Cy5. All cell lines except wild type express eGFP from the selectable marker cassette used in the knockouts. Fluorescence signal was visualized using a Beckman Coulter FC-500. c) 1×10^6^ wild type DT40 cells, non-green *Igl^KO^, Igh^KO^* cells and non-green *Igl^huVK^, Igh^huVH^* DT40 cells were labeled with FLUO-4-AM and incubated with 10 µg/ml goat-anti-chicken-IgM starting from the time point indicated by arrows. The change in fluorescence intensity was measured for a total of 300 sec using a Beckton Dickinson LSRII Fortessa. One of three representative experiments is shown. d) The same cell lines (DT40 wt grey, *Igl^KO^, Igh^KO^* red, *Igl^huVK^, Igh^huVH^* blue) were stained with goat-anti-human-kappa-RPE. Fluorescence was measured using a Beckman Coulter FC-500.

Cell lysates of the knock-out cells were prepared and analyzed by Western blot. The chicken Cµ was detected by a polyclonal anti-chicken-IgM antibody, and the chicken immunoglobulin light chain (IgL) was detected by a polyclonal anti-chicken-IgY antibody. Since DT40 cells express IgM and are not able to class switch, this antibody only detects IgL in DT40 cells. Wild-type DT40 cells show a double band at the expected molecular weight for the heavy chain at 74 kDa and for the light chain at 22 kDa. The *Igl^KO^* did not show any band with the IgY antibody but still shows a single band at 74 kDa for heavy chain. The *Igh^KO^* cells express chicken V_L_ but not V_H_. In the *Igl^KO^, Igh^KO^* cell line, the bands for heavy chain and light chain are absent (Fig. 2a). Cell surface staining of the immunoglobulin receptor with a monoclonal anti-chicken-Cµ antibody confirmed that the *Igl^KO^*, the *Igh^KO^* and the *Igl^KO^, Igh^KO^* cell lines are completely negative for surface IgM (Fig. 2b top row).

### Restoration of Surface IgM Expression by Insertion of Human V_K_ and V_H_ in Chicken Igl^KO^ and Igh^KO^ DT40 Cell Lines

**Figure 3:**
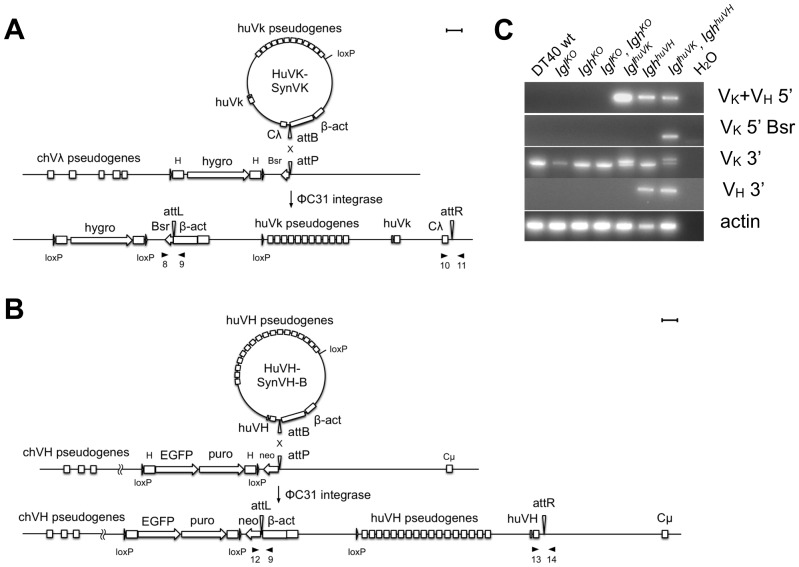
Integrase-mediated insertion of human V_L_ and V_H_ to replace the corresponding chicken genes. The *Igl^KO^* and *Igh^KO^* cell lines, as well as the *Igl^KO^, Igh^KO^* line, were used to insert human V_K_ or V_H_ into the chicken loci. Co-transfection of ΦC31 integrase and the shown a) huV_K_ or b) huV_H_ constructs resulted in recombination of the attP and attB sites leading to an insertion of the human V constructs and creating attL and attR sites. A β-actin promoter was integrated in front of the neomycin (neo) or blasticidin (Bsr) gene, and cells were selected with the indicated drug for stable integration of the huV_K_ or huV_H_. In the case of huV_K_ insertion into the chicken *Igl^KO^* single knockout, the selectable marker cassette was the same as for the heavy chain locus shown in b). c) To test for proper integration of the human genes, genomic DNA was isolated and a construct-specific PCR was performed with the indicated primers (primers are indicated by black arrowheads with primer orientation: VK+VH 5′, primers 12 and 9; VK 5′ Bsr, primers 8 and 9; VK 3′, primers 10 and 11; VH 3′, primers 13 and 14). Primers are placed on the 5′ and 3′ side of the integration showing the correct integration versus the knock out or wild type DT40 cell lines. β-actin was used as a quality control for the genomic DNA. Scale bar equals 1 kb.

Human V_K_ and V_H_ genes were inserted into the chicken V_L_ and V_H_ loci to produce chimeric antibodies consisting of human V regions and chicken constant regions. Since the constant regions are critical for a functional B cell receptor-signaling complex and for downstream effector functions *in vivo*, they must remain chicken sequence for optimal function. Insertion constructs included a human pseudogene array comprised of 7 to 17 designed pseudogenes. These are referred to as pseudogenes because they lack promoters and splice sites and are designed for gene conversion, not because they are related to the endogenous human VK and VH pseudogenes found in the human genome. Downstream of the pseudogene array, the huV_K_ constructs had a rearranged, functional human kappa variable region (consisting of V and J segments), the chicken J-C intron and the chicken lambda constant region gene followed by an attB site and the chicken β-actin promoter (Fig. 3a). The constructs for the huV_H_ insertion also had a pseudogene array and a rearranged, functional human V_H_ gene (consisting of V, D and J segments) followed by the attB site and a β-actin promoter (Fig. 3b). ΦC31 integrase-mediated recombination of the attB site in the inserting constructs and the attP site in the selectable marker cassette led to targeted integration into the immunoglobulin loci and placement of the β-actin promoter upstream of the neomycin or blasticidin resistance markers. Only cells with a targeted insertion into the immunoglobulin locus could be selected with neomycin or blasticidin.

To confirm the correct insertion on the 5′ side, PCR primers spanning from the β-actin promoter of the insertion vector into the neomycin or blasticidin resistance genes were used. The PCR from β-actin to neomycin gave a single band for the huV_K_ insertion into the chicken *Igl^KO^*, the huV_H_ into the chicken *Igh^KO^*, and the huV_H_ insertion into the chicken *Igl^KO^, Igh^KO^* (shown as VK+VH 5′ in Fig. 3c). PCR from β-actin into blasticidin gave a band only for the huV_K_ insertion into the *Igl^KO^, Igh^KO^* because blasticidin was used as a selectable marker (Fig. 3c).

Integration on the 3′ side was also confirmed by PCR. Primers for the V_L_ detected the non-rearranged wild-type allele in DT40 as well as the rearranged modified allele. Because of the resulting attR site after recombination of attP and attB, a double band was seen for correctly integrated clones (Fig. 3c). Correct integration of the huV_H_ on the 3′ side was confirmed with primers spanning from the huV_H_ into the chicken J-C intron (Fig. 3c).

Restoration of IgM expression in *Igl^huVK^* and *Igh^huVH^* single insertions and double *Igl^huVK^, Igh^huVH^* DT40 cell lines was analyzed. Lysates of PCR-positive clones were collected and Cµ or IgL were detected by Western Blot (Fig. 2a). The single huV_K_ insertion gave a strong double band at the expected molecular weight for light chain, comparable to wild type DT40 cells. The single huV_H_ insertion showed a single band around 74 kDa expected for heavy chain. The double insertion gave a band that is fainter than the wild type band at 75 kDa. The IgL blot shows a strong band for the huV_K_ integration at 25 kDa, a strong double band comparable to the wild type for the huV_H_ integration and a faint double band at the same size as the wild-type bands for the huV_K_-huV_H_ insertion.

Surface IgM expression was analyzed by flow cytometry using a monoclonal anti-chicken-Cµ antibody. All cells with a huV_K_ and wild type chicken heavy chain, as well as all cells with the double insertion *Igl^huVK^, Igh^huVH^*, showed surface IgM expression similar to wild type DT40 cells. In contrast, only a subpopulation (30.7%) of the cells with a huV_H_ insertion and wild type chicken light chain showed surface IgM expression (Fig. 2b bottom row). The *Igl^huVK^, Igh^huVH^* cells were also stained with a polyclonal anti-human-kappa antibody. Production of human V regions was supported by the shift of the *Igl^huVK^, Igh^huVH^* cells compared to wild type cells and *Igl^KO^, Igh^KO^* cells (Fig. 2d).

### A Human-chicken Chimeric IgM Receptor on Chicken DT40 Cells is able to Release a Calcium Signal after Cross-linking

EGFP-positive *Igl^KO^, Igh^KO^* cells, as well as *Igl^huVK^, Igh^huVH^* cells, were transiently transfected with CAG-Cre to remove the selectable marker cassettes by Cre-lox recombination. After 72 h 35% to 45% eGFP-negative cells were observed. Limiting dilution was performed and clonal non-green populations of double knock-out cells and double insertion cells were grown and analyzed for calcium signaling. Cells were labeled with FLUO-4 calcium-dependent dye and IgM surface receptors were cross-linked using a polyclonal anti-chicken-IgM antibody. After cross-linking, an increase in fluorescence was measured for DT40 wild-type and *Igl^huVK^, Igh^huVH^* cells by flow cytometry. The increase in fluorescence was higher for *Igl^huVK^, Igh^huVH^* cells compared to DT40 wild type cells. By contrast, the addition of polyclonal anti-chicken IgM antibody to *Igl^KO^, Igh^KO^* knock out cells did not change fluorescence (Fig. 2c).

### Creation of Diversity in huV_K_ and huV_H_ Sequences by Gene Conversion in DT40 Cells

**Figure 4:**
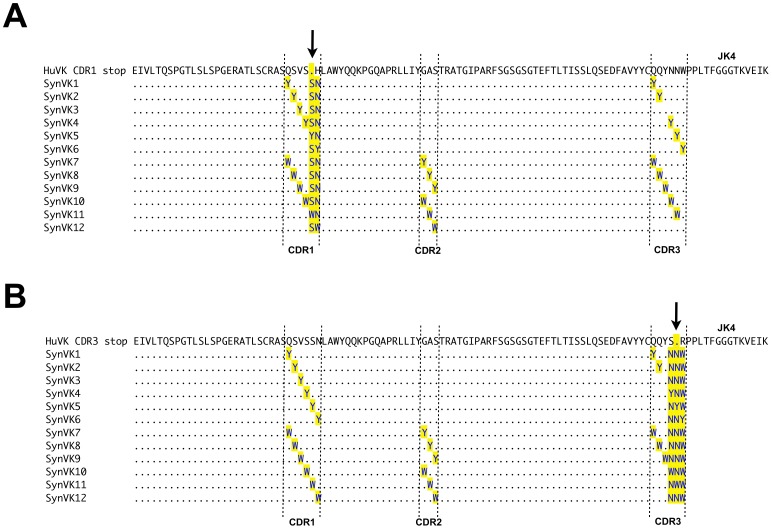
Alignment of the SynVK-12 pseudogene array with functional huV_K_. The human SynVK-12 pseudogenes SynVK1 to SynVK12 were aligned with functional huV_K_ using Lasergene (DNAStar Inc.,Madison, USA). Complementarity determining regions (CDR) are marked by upright dashed lines. The arrow indicates the position of the HpaI site and stop codon inserted in a) CDR1 or b) CDR3.

**Figure 5:**
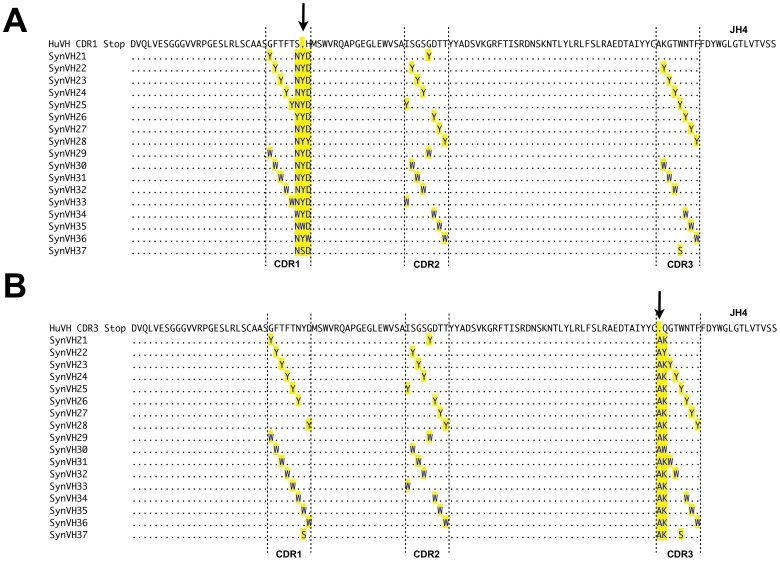
Alignment of the SynVH-B pseudogene array with functional huV_H_. The human SynVH-B pseudogenes SynVH21 to SynVH37 were aligned with functional huV_H_ using Lasergene (DNAStar Inc.,Madison, USA). CDRs are marked by upright dashed lines. The arrow indicates position of the HpaI site and stop codon inserted in a) CDR1 or b) CDR3.

**Figure 6:**
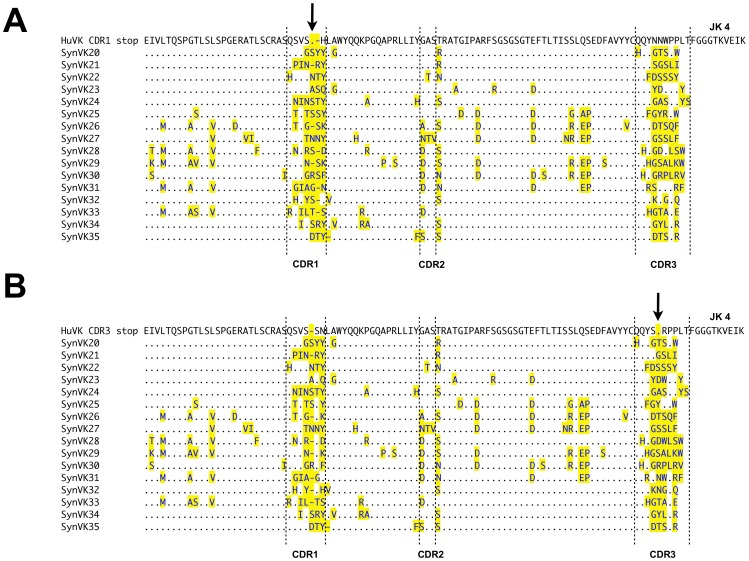
Alignment of the SynVK-C pseudogene array with functional huV_K_. The human SynVK-C pseudogenes SynVK20 to SynVK35 were aligned with functional huV_K_ using Lasergene (DNAStar Inc.,Madison, USA). CDRs are marked by upright dashed lines. The arrow indicates the position of the HpaI site and stop codon inserted in a) CDR1 or b) CDR3.

Human pseudogene arrays containing synthetically-designed V regions (SynV genes) were integrated upstream of the functional huV_K_ and huV_H_ in DT40 cells. Two different types of SynV pseudogenes were designed. In the SynVK-12 and SynVH-B arrays, each of the pseudogenes contained complementarity determining regions (CDRs) with individual tyrosine or tryptophan substitutions at every position in each CDR (Figs. 4 and 5). This array design was based on evidence that minimalist CDRs consisting of appropriately spaced tyrosine and tryptophan residues are capable of producing high affinity antigen-specific antibodies [33], [34]. In contrast, the SynVK-C pseudogenes consisted of naturally occurring human CDR sequences from expressed, full-length V regions (Fig. 6), which provides a larger pool of diverse sequences with which to mutate the functional V. For some of the SynVK-C pseudogenes, the framework regions between the CDRs were different from the frameworks used in the functional huV_K_, in order to provide further sequence diversity.

To select for gene-converted sequences by PCR, a reversion assay was designed. A HpaI restriction site, containing a stop codon, was included in CDR1 or CDR3 of the transcribed huV_K_ and huV_H_ transgenes. These constructs were identical to those used for the expression analysis in Fig. 2 except for the HpaI site and stop codon. DT40 cells were tranfected with these constructs, resistant clones were confirmed by PCR, and the huV_K_ and huV_H_ sequences were analyzed after growing these cells at least four weeks to allow accumulation of mutations. Genomic DNA was isolated and digested with HpaI. Only huV mutations that eliminated the HpaI site (either by gene conversion or other type of mutation) could then serve as template in the PCR reaction. To ensure that intact, unmutated huV amplicons were not cloned, the PCR product was digested again with HpaI, cloned and sequenced. In most cases we used the unsorted bulk culture to analyze all of the Hpa-resistant sequences, but in the case of the SynVK-12 array with a stop in CDR1 (Fig. 4a) we first sorted the cells based on surface IgM expression (Fig. 2b, *Igl^huVK-Stop^*).

**Figure 7:**
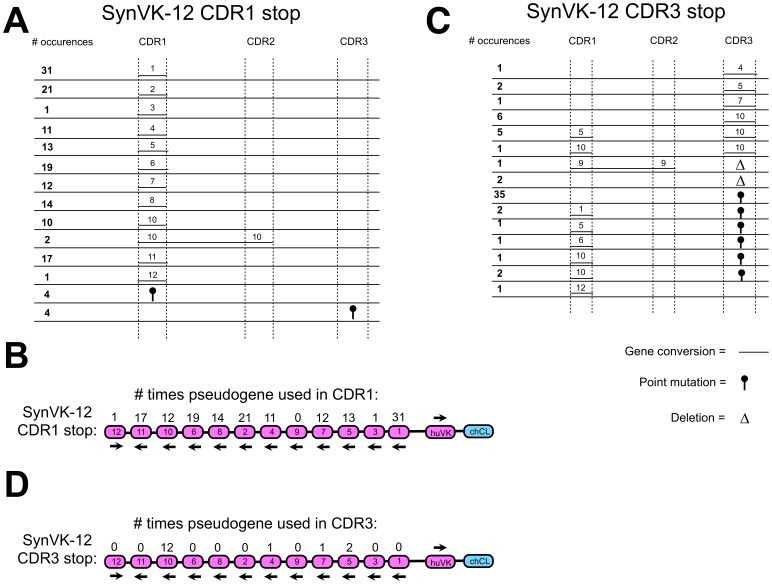
Creating huV_K_ diversity by gene conversion using a human light chain pseudogene array with single amino acid changes in the CDRs. The chicken V_L_ was replaced by a huV_K_ including 12 human synthetic pseudogenes with single amino acid changes in the CDR sequences (the SynVK-12 array). The inserted huV_K_ had a stop codon and a HpaI restriction enzyme site in CDR1 or CDR3 so that there was no IgM expression unless the stop codon was repaired due to gene conversion. DT40 cells were cultured for four weeks after insertion, and afterwards analyzed by sequencing for possible gene conversion events, deletions and point mutations. Gene conversion events for the stop in CDR1 are shown in a) and b). Gene conversion events for the stop in CDR3 are shown in c) and d). In diagrams a) and c), the length of the line showing the gene conversion events corresponds with the actual length of the gene conversion event observed, and the number above the line indicates the pseudogene name. Frequency of every event is shown in bold at left. The arrows under the pseudogenes and the functional V in c–d) show the orientation of the genes. Every change in the parental sequence was counted as an individual event.

In the SynVK-12 construct, most pseudogenes were used frequently for gene conversion of CDR1, except for pseudogenes 3, 9 and 12, which were only used once or not at all (Fig. 7). There was a bias towards pseudogene 1, which is the most proximal to the functional huVK (Fig. 7b). Only two long gene conversion events extending from CDR1 to CDR2 were observed (Fig. 7a). For CDR3, a high frequency of point mutations was observed: 42 point mutations out of 62 sequences analyzed for the SynVK-12 array (Fig. 7c). Only pseudogenes 4, 5, 7 and 10 were used for gene conversion of CDR3 (Fig. 7d). Also, some gene conversion events for CDR1 and CDR2 independent from the selection for CDR3 were detected (Fig. 7c).

**Figure 8:**
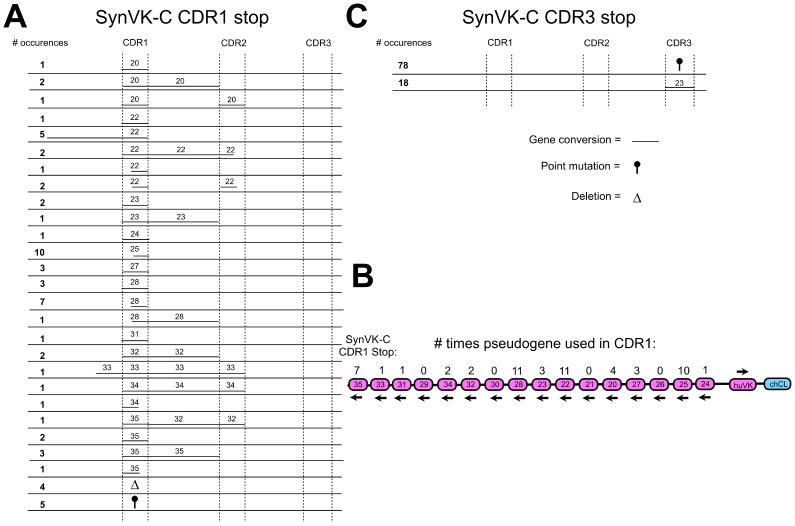
Creating huV_K_ diversity by gene conversion using a human light chain pseudogene array with naturally occurring CDRs, AID optimization and framework changes. The chicken V_L_ was replaced by a huV_K_ including 16 human synthetic pseudogenes containing naturally occurring CDR sequences from an EST database (NCBI), the SynVK-C array. In addition the pseudogenes and the functional V_L_ were AID optimized. Some of the pseudogenes also differ in their framework regions compared to the functional huV_K_. The inserted huV_K_ had a stop codon and a HpaI restriction enzyme site in CDR1 or CDR3 so that there was no IgM expression unless the stop codon was repaired due to gene conversion. DT40 cells were cultured for four weeks after insertion and afterwards analyzed by sequencing for possible gene conversion events, deletions and point mutations. Gene conversion events for the stop in CDR1 are shown in a) and b). Gene conversion events for the stop in CDR3 are shown in c). The length of the line showing the gene conversion events corresponds with the actual length of the gene conversion event observed. Frequency of every event is shown in bold at left. The arrows under the pseudogenes and the functional V in b) show the orientation of the genes. Every change in the parental sequence was counted as individual event.

In the SynVK-C construct, the sequence of the functional huVK was modified to include hotspots for activation-induced cytidine deaminase (AID), and the pseudogenes were also similarly modified, to retain sequence homology with the functional V. Most SynVK-C pseudogenes were used for gene conversion of CDR1 except for pseudogenes 21, 26, 29 and 30 (Fig. 8b). Long events from CDR1 to CDR2 were observed for the SynVK-C array (Fig. 8a). For CDR3, 78 out of 96 sequences showed point mutations and no recognizable gene conversion events. In 18 of the sequences, gene conversion was detected for CDR3 with the SynVK-C array, yet in every case it came from pseudogene 23 (Fig. 8c).

**Figure 9:**
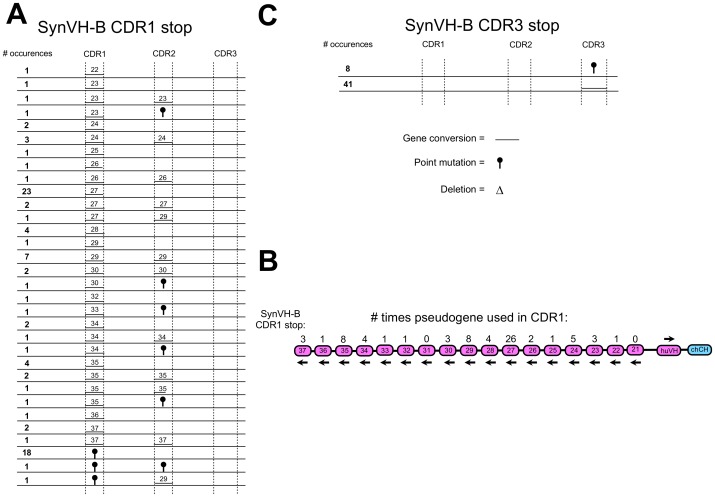
Creating huV_H_ diversity by gene conversion using a human heavy chain pseudogene array with single amino acid changes in the CDRs. The chicken V_H_ was replaced by a huV_H_ including 17 human synthetic pseudogenes with single amino acid changes in the CDR sequences (the SynVH-B array). The inserted huV_H_ had a stop codon and a HpaI restriction enzyme site in CDR1 or CDR3 so that there was no IgM expression unless the stop codon was repaired due to gene conversion. DT40 cells were cultured for four weeks after insertion and afterwards analyzed by sequencing for possible gene conversion events, deletions and point mutations. Gene conversion events for the stop in CDR1 are shown in a) and b). Gene conversion events for the stop in CDR3 are shown in c). No pseudogene name is indicated over the line in CDR3 in c) because the sequences could have been derived from every pseudogene except 22 and 30. The length of the line showing the gene conversion events corresponds with the actual length of the gene conversion event observed. Frequency of every event is shown in bold. Every change in the parental sequence was counted as individual event.

Analysis of gene conversion of the huV_H_ gene in DT40 cells revealed that the SynVH-B array was used very efficiently for gene conversion in CDR1 (Fig. 9). All pseudogenes except 21 and 31 were used for gene conversion in CDR1. An apparent bias toward pseudogene 27 was observed (Fig. 9b). Long events going from CDR1 to CDR2 were detected (Fig. 9a). Analysis of the sequences for CDR3 showed only one sequence pattern (Fig. 9c). It was not possible to determine the origin of this gene conversion event since all pseudogenes except 22 and 30 have the same sequence at the beginning of CDR3 at the position where the HpaI site is located. Point mutations were also observed.

**Figure 10:**
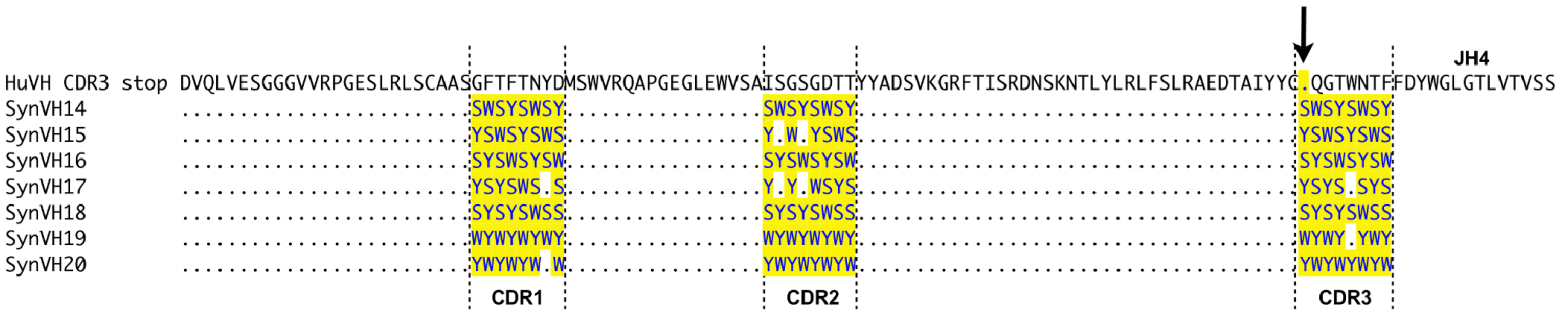
Alignment of the SynVH-A7 pseudogene array with functional huV_H_. The human SynVH-A7 pseudogenes SynVH14 to SynVH20 were aligned with functional huV_H_ using Lasergene (DNAStar Inc.,Madison, USA). CDRs are marked by upright dashed lines. The arrow indicates the position of the HpaI site and stop codon inserted in CDR3.

**Figure 11:**
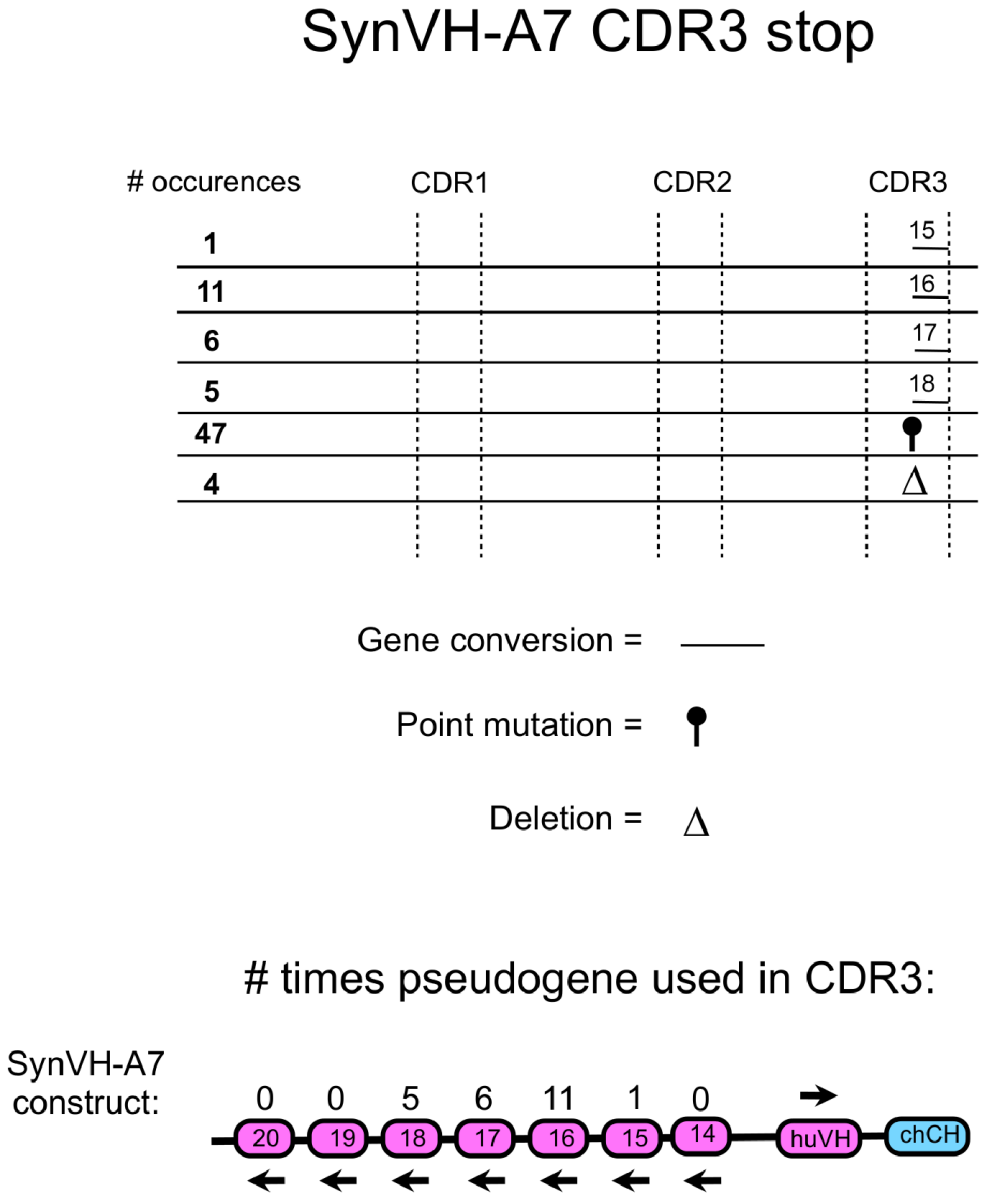
Creating huV_H_ diversity by gene conversion using a human heavy chain pseudogene array with fully synthetic CDRs. The chicken V_H_ was replaced by a huV_H_ including 7 human pseudogenes with fully synthetic CDR sequences (the SynVH-A7 array). The inserted huV_H_ had a stop codon and a HpaI restriction enzyme site in CDR3 so that there was no IgM expression unless the stop codon was repaired due to gene conversion. DT40 cells were cultured for four weeks after insertion and afterwards analyzed by sequencing for possible gene conversion events, deletions and point mutations. Gene conversion events for the stop in CDR3 are shown. The length of the line showing the gene conversion events corresponds with the actual length of the gene conversion event observed. Frequency of every event is shown in bold. Every change in the parental sequence was counted as individual event.

To analyze if it is possible to generate more diversity for CDR3 in huV_H_, an array with seven pseudogenes called SynVH-A7 was used. Every amino acid position in each CDR in the SynVH-A7 array was substituted with serine, tyrosine or tryptophan (Fig. 10). Analyzing gene conversion events from this array in CDR3 showed 23 partial gene conversion events replacing the HpaI site, out of 91 sequences. 4 different pseudogenes out of seven were used (Fig. 11).

## Discussion

Here we describe a novel cell-based system to diversify exogenous genes of interest, and applied it to the diversification of human variable region genes. The system was used to validate the constructs that will be inserted into a line of transgenic chickens expressing chimeric immunoglobulins. A two-step approach to replace the endogenous chicken V regions with human V regions was taken: first, knockouts of the chV_L_ and chV_H_, both individually and together, were created and analyzed, followed by site-directed insertion of functional human Vs in conjunction with synthetic human pseudogene arrays. This is the first description of a knockout of the functional heavy chain V in chicken cells.

The targeting vectors used to knock out the chicken Ig loci inserted selectable marker cassettes and attP sites into the chicken *Igl* and *Igh* loci. By co-transfection with φC31 integrase and an insertion cassette containing an attB site, complex constructs can now be efficiently inserted into the Ig loci [16], [35]. The *Igl^KO^, Igh^KO^* knockout cell line thus represents a “universal acceptor” that can be harnessed to diversify any given exogenous gene or pair of genes by gene conversion [24]. The design of the pseudogene arrays dictates how genes can be diversified: which domains or regions of the target genes are to be mutated, and which residues are available to the gene conversion process with which to mutate them.

While it was possible to restore surface IgM expression by expressing huV_K_ in combination with the chV_H_ in the *Igl^huVK^* cell line, the converse did not lead to surface IgM expression on every cell. Western blot analysis suggested that the huV_H_ and chV_L_ do not pair efficiently, leading to protein degradation in the cells. Efficient pairing was restored by inserting both huV_K_ and huV_H_ into the same cells. Since the chicken constant regions are the same in all the insertion cell lines, the difference in the functional V enabled the assembled IgM receptor to express on the surface at levels indistinguishable from wild-type DT40 cells. The difference in the molecular weight of the chimeric heavy chain in the double insertion cell line compared to wild type chicken heavy chain may be explained by a different glycosylation pattern of the huV_H_ compared to the chV_H_. After cross-linking the surface IgM with a polyclonal anti-chicken-IgM antibody a strong calcium signal was detected indicating that the human-V-chicken-constant-region chimeric receptor assembled correctly and was functional. The calcium release observed in *Igl^huVK^, Igh^huVH^* cells was stronger than the signal detected in wild-type cells, as shown by the higher proportion of cells that shift upon stimulation. One possible explanation for this difference is that we have inadvertently selected a high-signaling variant after 5 rounds of clonal selection to produce the *Igl^huVK^, Igh^huVH^* cells (2 knockouts, 2 insertions, and limiting dilution after Cre transfection).

Rates of productive gene conversion that repaired the HpaI/stop codon and restored surface IgM were between 1 and 3% for the huV_K_ and the huV_H_ with a stop codon in CDR1, and approximately 0.5% for the stop codon in CDR3, after culturing the cells for four weeks (Fig. 2b and data not shown). Gene conversion of endogenous V genes occurs at a rate of 1% in DT40 cells [21], consistent with the rates seen for our transgenes. Observed frequencies *in vivo* appear much higher, with 3–7 gene conversion events found in each sequence analyzed from bursal B cells at 3 weeks of age [36], suggesting that we will obtain much higher frequencies in our transgenes *in vivo*. Analysis of gene conversion in surface IgM-restored SynVK-12 cells showed that two pseudogenes were rarely used for CDR1, indicating that the sequences in those pseudogenes were not compatible with expression (Fig. 7b). All other analysis was performed on unsorted cells to capture all of the potential sequence diversity regardless of expressability. The observed biases in pseudogene usage in unsorted cells cannot be easily explained. Neither proximity to the functional V nor sequence homology correlate with frequency. Sequence analysis of the huV_K_ and huV_H_ showed that the lower frequency of gene conversion observed in CDR3 is consistent with previous data published for the chicken V_L_ in DT40 cells [37]. In contrast to endogenous chicken B cells [36], DT40 cells show a strong bias towards point mutations and short gene conversion events in CDR3 [18].

The frequencies of point mutations in CDR3 for most of the arrays were correspondingly high: the SynVK-12 and SynVK-C arrays showed point mutations of the huV_K_ CDR3 at a rate of 67% and 81% of the sequences analyzed, respectively, and the SynVH-A7 array showed a rate of 63% for huV_H_. In contrast, the SynVH-B array showed point mutations at a lower rate of 16%. This might be due to the design of CDR3 in the pseudogenes of the SynVH-B array. CDR3 of the SynVH-B pseudogenes 21,22, 29, 30 and 33 is identical with the sequence downstream of the HpaI site in the functional huV_H_ (Fig. 5), and this sequence homology may have increased the gene conversion frequency with a concomitant reduction in point mutation frequency in the sequences analyzed. Reynaud et al. have shown that for CDR3, the preferred pseudogenes were those that display homology on the 3′ side towards CDR3 in the functional V [36].

For the purpose of increasing gene conversion efficiency, the sequence of the functional V_K_ for the SynVK-C array and the SynVK-C pseudogenes was AID optimized. Without changing the protein sequence, the nucleotide sequence was changed where possible to WGCW [38]. The analysis of the CDR1 HpaI-stop codon restriction site reversion assay showed a significant increase towards long gene conversion events compared to the SynVK-12 array without AID optimization. With the SynVK-C array, 24% of the gene conversion events for CDR1 were long events, whereas for SynVK-12, only 1% of the gene conversion events for CDR1 were long events extending to CDR2. Interestingly, the enhanced gene conversion rate was not seen for SynVK-C in CDR3.

Taken together, these data demonstrate that the diversification machinery of DT40 cells can be harnessed to accomplish in vitro molecular evolution. It will be interesting to compare sequences obtained from *in vivo* studies with these same human transgenes to determine the extent to which gene conversion and somatic hypermutation diversify the human V regions both in the pre-immune repertoire and in the sequences of antigen-specific antibodies. It remains to be seen whether the level of diversity introduced by gene conversion and point mutations in our transgenes will lead to antibodies of high affinity in transgenic birds. The lack of multiple gene families and combinatorial diversity of these human transgenes should not be a limitation, since normal chickens produce a highly diverse pre-immune repertoire from single functional V regions by gene conversion. The method provides the ability to circumscribe the boundaries of the canonical variants through the design of the pseudogene array. These results illustrate the utility of DT40 cells for testing and validating human immunoglobulin constructs that will be used to create a transgenic chicken platform for the discovery and development of novel human antibody therapeutics.
